# Clinical expert consensus document on drug-coated balloon for coronary artery disease from the Japanese Association of Cardiovascular Intervention and Therapeutics

**DOI:** 10.1007/s12928-023-00921-2

**Published:** 2023-02-27

**Authors:** Takashi Muramatsu, Ken Kozuma, Kengo Tanabe, Yoshihiro Morino, Junya Ako, Shigeru Nakamura, Kyohei Yamaji, Shun Kohsaka, Tetsuya Amano, Yoshio Kobayashi, Yuji Ikari, Kazushige Kadota, Masato Nakamura, The Task Force of the Japanese Association of Cardiovascular Intervention, Therapeutics (CVIT)

**Affiliations:** 1 Department of Cardiology, Cardiovascular Center, Fujita Health University Hospital, 1-98 Dengaku, Kutsukake, Toyoake, Aichi 470-1192 Japan; 2 Division of Cardiology, Teikyo University Hospital, Tokyo, Japan; 3 Division of Cardiology, Mitsui Memorial Hospital, Tokyo, Japan; 4 Division of Cardiology, Department of Internal Medicine, Iwate Medical University, Iwate, Japan; 5 Department of Cardiovascular Medicine, Kitasato University School of Medicine, Sagamihara, Japan; 6 Cardiovascular Center, Kyoto Katsura Hospital, Kyoto, Japan; 7 Department of Cardiovascular Medicine, Kyoto University, Kyoto, Japan; 8 Department of Cardiology, Keio University School of Medicine, Tokyo, Japan; 9 Department of Cardiology, Aichi Medical University, Nagakute, Japan; 10 Department of Cardiovascular Medicine, Chiba University Graduate School of Medicine, Chiba, Japan; 11 Department of Cardiology, Tokai University School of Medicine, Isehara, Japan; 12 Department of Cardiovascular Medicine, Kurashiki Central Hospital, Kurashiki, Japan; 13 Division of Cardiovascular Medicine, Toho University Ohashi Medical Center, Tokyo, Japan

**Keywords:** Drug-coated balloon, Coronary artery disease, Percutaneous coronary intervention, Restenosis

## Abstract

Drug-coated balloon (DCB) technology was developed to deliver the antiproliferative drugs to the vessel wall without leaving any permanent prosthesis or durable polymers. The absence of foreign material can reduce the risk of very late stent failure, improve the ability to perform bypass-graft surgery, and reduce the need for long-term dual antiplatelet therapy, potentially reducing associated bleeding complications. The DCB technology, like the bioresorbable scaffolds, is expected to be a therapeutic approach that facilitates the “leave nothing behind” strategy. Although newer generation drug-eluting stents are the most common therapeutic strategy in modern percutaneous coronary interventions, the use of DCB is steadily increasing in Japan. Currently, the DCB is only indicated for treatment of in-stent restenosis or small vessel lesions (< 3.0 mm), but potential expansion for larger vessels (≥ 3.0 mm) may hasten its use in a wider range of lesions or patients with obstructive coronary artery disease. The task force of the Japanese Association of Cardiovascular Intervention and Therapeutics (CVIT) was convened to describe the expert consensus on DCBs. This document aims to summarize its concept, current clinical evidence, possible indications, technical considerations, and future perspectives.

## Introduction

Drug-eluting stent (DES) technologies have significantly reduced the risk of restenosis and stent thrombosis, establishing the safety and efficacy of percutaneous coronary interventions (PCI) for the treatment of obstructive coronary artery diseases (CAD) [1–4]. An inherent limitation of the metallic stent is the presence of a foreign material within the native coronary artery, which can cause vascular inflammation, hypersensitivity, neoatherosclerosis and subsequent stent thrombosis [5, 6]. It should be emphasized that the late luminal enlargement and restoration of vasomotor function are both compromised by the permanent metallic cage [7, 8]. A drug-coated balloon (DCB) is an attractive alternative to DES in that it delivers antiproliferative drugs (e.g., paclitaxel or sirolimus) directly to the vessel wall via a lipophilic matrix, eliminating the need for a permanent carrier such as a metallic prosthesis and/or a durable polymer. Safety and efficacy of this technology have been studied primarily in patients with in-stent restenosis (ISR) or de novo small vessel lesions, but wider clinical indications are being considered. In this scenario, we need to recognize the importance of lesion assessment and technical issues, as well as the limitations of this technology in order to provide the best clinical practice for patients. Herein, the task force of the Japanese Association of Cardiovascular Intervention and Therapeutics (CVIT) was convened to describe the expert consensus on DCBs. This document aims to summarize its concept, current clinical evidence, possible indications, technical considerations, and future perspectives.

## Drug-coated balloon technologies

A balloon catheter, a highly lipophilic drug, and a coating matrix that regulates local drug delivery to the vessel wall comprise the DCB. In contrast to the stent-based technologies, the DCB is able to deliver the drug uniformly to the vessel wall [9]. Paclitaxel is primarily used as an antiproliferative drug, and a specific balloon coating containing a contrast medium as an excipient reduced neointimal hyperplasia in a porcine coronary overstretch model [10]. A rapamycin analogue (i.e., sirolimus) was recently tested in human coronary arteries. These two predominant antiproliferative drugs act differentially with tissue. Paclitaxel absorbs quickly, localizes in subintimal space, and partitions significantly in adventitia, whereas sirolimus absorbs slowly and spreads throughout entire artery where it dilutes down to subtherapeutic levels [11]. A major drawback of sirolimus and its derivatives is generally the poorer transfer rate compared to paclitaxel [12]. Due to reversible binding to the mammalian target of rapamycin receptor (mTOR), this poses a technical challenge in maintaining drug permeation in tissue [13]. Crystalline coating sustains higher and longer drug concentrations in tissue compared to amorphous coating [14]. Other novel ideas have been proposed, such as micro-reservoir or nano-technology for balloon-based local drug administration (Table 1). Given such significant differences in antiproliferative drugs and their doses, release kinetics, and tissue concentrations, it would be premature to expect a “class effect” among various DCB technologies [15]. Nevertheless, the recent randomized AGENT Japan trial demonstrated comparable clinical safety and efficacy within 6 months between the two DCBs with different paclitaxel doses and excipients [16].

**Table 1:** Commercially available or under investigation drug-coated balloons for coronary artery diseases

Device	Company	Drug	Dose (μg/mm^2^)	Additive	Availability in Japan
SeQuent Please Neo	B. Braun	Paclitaxel	3.0	Iopromide	Yes
Agent	Boston Scientific	Paclitaxel	2.0	Acetyl tributyl citrate	Yes
Prevail	Medtronic	Paclitaxel	3.5	Urea	No
Pantera Lux	Biotronik	Paclitaxel	3.0	*n*-Butyryl citrate	No
RESTORE	Cardionovum	Paclitaxel	3.0	Shellac	No
Elutax SV	Aachen Resonance	Paclitaxel	2.2	None	No
Magic Touch	Concept Medical	Sirolimus	1.3	Phospholipid	No
Selution	Med Alliance	Sirolimus	1.0	Micro-reservoirs	No
Virtue	Caliber Therapeutics	Sirolimus	N/A	Nanoparticles	No
SeQuent SCB	B. Braun	Sirolimus	4.0	Crystalline	No

## Rationale of “stentless” or “leave nothing behind” strategy using DCB

One possible adverse outcome of coronary lesions treated with metallic DES is the development of in-stent neointimal tissue, which the antiproliferative drug slows or delays. It is overly optimistic to regard this phenomenon as benign because neointimal tissue can become atherosclerotic, degenerate into a vulnerable plaque, and eventually rupture inside the metallic cage (i.e., neoatherosclerosis) [6].

A stiff metallic stent also alters vessel geometry and biomechanics, which can lead to long-term flow disturbances and chronic irritation, as well as the risk of late strut fractures, which can lead to restenosis and adverse clinical events [17, 18]. From a physiological perspective, the absence of a rigid metallic cage facilitates the restoration of vasomotor function, adaptive shear stress, and late luminal enlargement [7, 19]. At mid-term follow-up ranging 6–8 months, serial angiographic or optical coherence tomography (OCT) studies revealed that 40–56% of lesions treated with DCB had late luminal enlargement [16, 20, 21]. In contrast to the metallic DES, DCB would have no triggers for thrombosis, such as uncovered struts or durable polymers. The absence of foreign materials can reduce the risk of very late stent failure, improve the ability to perform bypass-graft surgery, and reduce the need for long-term dual antiplatelet therapy (DAPT), potentially reducing associated bleeding complications. Given the significant impact of suggestive metal allergy on recurrent ISR, the DCB rather than the DES would be a reasonable therapeutic approach for patients with suspected metal allergy [22].

It should also be noted that this technology may be relevant to East-Asian populations (e.g., Japanese) who have different cultures than Western countries in that they do not want to mutilate their natural bodies with medical devices or even surgical incisions that will last their entire lifetime.

## Current usage of DCB in Japan

**Fig. 1:**
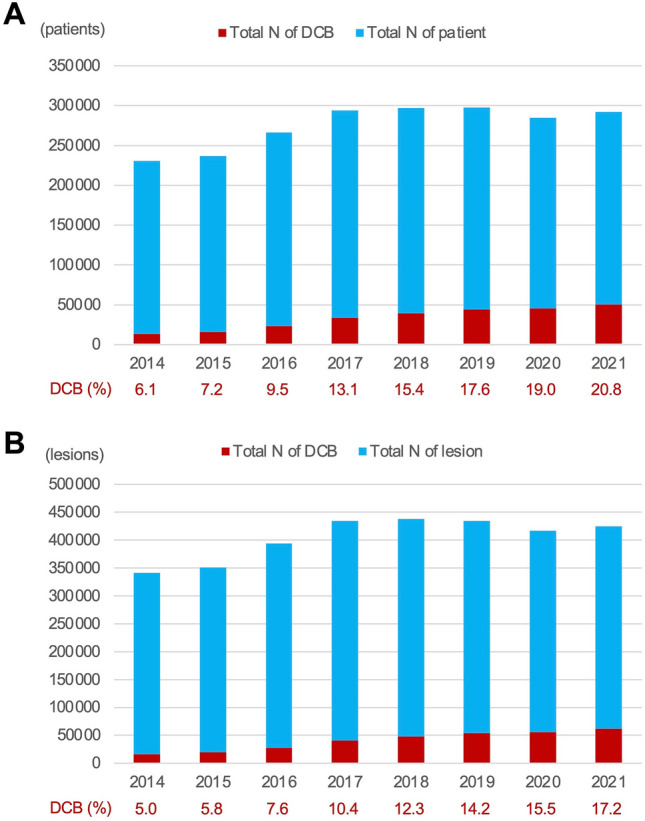
Change in the number and proportion of drug-coated balloon usage in the J-PCI registry (2014–2021). **a** Per patient analysis and **b** per lesion analysis. The SeQuent Please® and the SeQuent Please Neo® (B. Braun, Melsungen, Germany) paclitaxel-coated balloons were clinically available in Japan during the period. DCB = drug-coated balloon

Figure 1 depicts the change in the number and proportion of DCBs used over time in the nationwide J-PCI registry sponsored by the CVIT [23]. Clearly, the number and the proportion of DCB usage during PCI have shown an increasing trend in Japan (*p* for trend < 0.01). In 2021, DCB was used in 20.8% of 241,661 patients and 17.2% of 362,468 lesions. It is assumed that this is due to the accumulation of experience in use and confirmation of efficacy in actual clinical practice.

## Clinical indications for DCB use

### In-stent restenosis (ISR)

ISR has been considered as one of the “best” target for the DCB because it can avoid multiple layers of metallic stents. Clinical safety and efficacy of the DCB for ISR lesions have been thoroughly studied, and current Japanese and Western guidelines offer consistent recommendations (class I, level of evidence A) [24, 25]. Table 2 summarizes randomized trials comparing DCB and DES for ISR. It is important to understand the substantial difference of ISR between bare-metal stents (BMS) and DES. The BMS-ISR is generally characterized by excessive neointimal proliferation (i.e., hyperplasia), whereas the DES-ISR is more complicated because it can be perceived as a result of antiproliferative drugs’ insufficient effect or failure to function (i.e., late neoatherosclerosis). Indeed the DES-ISR was only independent predictor of target lesion revascularization (TLR) in Japanese pre- and post-marketing study for the SeQuent Please® DCB [26]. Intracoronary imaging techniques, preferably OCT, would be recommended to identify the underlying causes of ISR (class IIa, level of evidence C) [24].

**Table 2:** Randomized controlled trials comparing drug-coated balloon and drug-eluting stent for in-stent restenosis

Trial (year)	Comparator (N)	Primary endpoint	Clinical F/U (months)	Results
BMS-ISR				
RIBS V (2014)	EES (189)	MLD at 9 months (angio)	12	MLD: DCB 2.01 mm vs. EES 2.36 mm (*p* < 0.01)
				MACE: DCB 9% vs. EES 6% (*p* = 0.65)
SEDUCE (2014)	EES (50)	Uncovered strut (%) at 9 months (OCT)	12	DCB 1.4% vs EES 3.1% (*p* = 0.03)
				Binary restenosis: DCB 9.1% vs. EES 0% (*p* = 0.15)
DES-ISR				
RIBS IV (2015)	EES (309)	MLD at 9 months (angio)	12	DCB 2.03 mm vs. EES 1.80 mm (*p* < 0.01)
				MACE: DCB 20.1% vs. EES 12.3% (*p* = 0.04)
RESTORE (2018)	EES (172)	In-segment LLL (angio)	12	DCB 0.15 mm vs. EES 0.19 mm (*p* = 0.54)
				MACE: DCB 7.0% vs. EES 4.7% (*p* = 0.51)
Mixed-ISR				
DARE (2018)	EES (278)	MLD at 6 months (angio)	12	DCB 1.72 mm vs. EES 1.84 mm (*p* = 0.02)
				MACE: DCB 10.9% vs. EES 9.2% (*p* = 0.66)

ISR, in-stent restenosis; BMS, bare-metal stent; DES, drug-eluting stent; EES, everolimus eluting stent; DCB, drug-coated balloon; MLD, minimum lumen diameter; MACE, major adverse cardiac events; LLL, late lumen loss

### Small vessels

Small vessel disease is usually defined as < 3.0 mm in reference vessel diameter. Stent or scaffold implantation in such a small vessel may be disadvantageous when compared to the implantation in a large vessel because late lumen loss occupies a greater percentage of the respective vessel diameter, resulting in higher rates of ISR and adverse events [27]. In the randomized BASKET-SMALL 2 trial, a paclitaxel-iopromide-coated DCB was found to be non-inferior to the second generation DES in terms of a composite of cardiac death, non-fatal myocardial infarction, or target vessel revascularization (TVR) at 12 months [28]. Furthermore, the RESTORE SVD China trial found that the DCB was non-inferior to the Resolute Integrity® DES in terms of percentage diameter stenosis at 9 months, and had comparable target lesion failure (TLF) rates at 1 year [29]. These findings may support the use of DCB as an alternative to DES in small vessel diseases, but only when lesion preparation is sufficiently accomplished.

### Possible de novo large vessel lesions

Despite growing and encouraging scientific evidences of DCB in de novo lesions with large reference vessel diameters (≥ 3.0 mm), randomized comparison with DES for this indication remains limited. In the DEBUT trial, individuals with a high bleeding risk (HBR) were compared between the DCB and the BMS. Within 9 months, there was no acute vessel closure and only 1% of major adverse cardiac events in the DCB group, demonstrating its superiority to the BMS [30]. In the PEPCAD NSTEMI trial, non-inferiority of a paclitaxel-iopromide-coated DCB regarding TLF at 9 months was evident compared to the metallic stents (i.e., BMS or DES) [31]. However, in the DCB group, 85% of patients received only DCB, while 15% received additional stent implantation. This result highlights a possibility of “bail-out” stenting being unavoidable even in a certain amount of cases who would prefer DCB-only strategy.

For this possible indication of DCB, the CVIT recently issued an official statement (https://www.cvit.jp/_assets/documents/news/2023/0104.pdf). Briefly, the use of DCB for de novo large vessel lesions will be possible subject to registration in a real-world all-comers ALLIANCE registry. The statement also offered the following conditions; (1) patients having HBR or those for whom long-term antiplatelet therapy is considered undesirable; or (2) lesions for which clinical effectiveness of DES is not well established (e.g., ostial circumflex or jailed side branch).

Clinical implications of DCB for specific patient or lesion subset are discussed in the following sub-headings.

#### Bifurcation lesions

Bifurcation is still a challenging lesion subset and it is responsible for approximately 20% of cases undergoing PCI [32, 33]. The most widely accepted approach to date is single cross-over stenting in the main vessel, with side branch balloon dilatation or provisional stenting as needed [25]. Following ostial side branch balloon dilatation, subsequent DCB significantly reduced angiographic late lumen loss (0.13 mm vs. 0.51 mm) and binary restenosis at 9 months (6% vs. 26%) compared to no additional treatment [34]. A similar finding was confirmed in a meta-analysis of 349 cases comparing conventional balloon and DCB for side branch outcomes [35]. Based on these results, DCB would be a preferable approach to the side branch when the DES was deployed in the main vessel. The DCB-only approach to bifurcation is appealing because it avoids carina shift, but it is more technically challenging. In this scenario, sequential DCB dilatation following optimal lesion preparation is recommended because kissing DCB dilatation may develop coronary dissection or perforation. DCB clearly provides an advantage by reducing the number of stent layers in patients with bifurcation restenosis, particularly when the index PCI used two-stent techniques [36]. The recent European Bifurcation Club (EBC) consensus document, however, does not support systematic DCB use in de novo bifurcation lesions due to a lack of conclusive evidence [37].

**Fig. 2:**
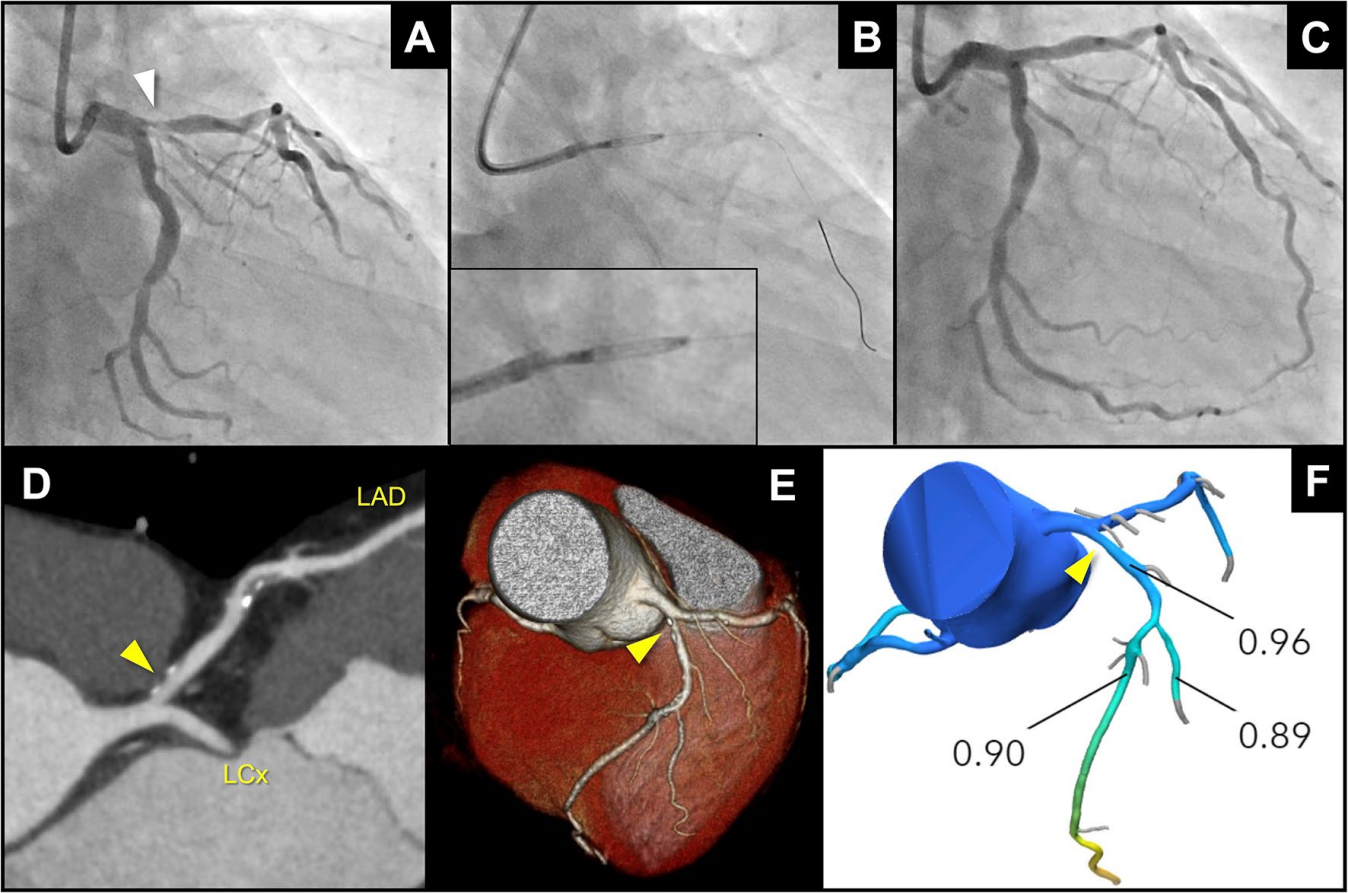
A case treated with DCB-only strategy using directional coronary atherectomy for ostial left anterior descending lesion. A 65-year-old man presenting with chronic coronary syndrome. **a** Pre-procedural angiogram revealed a focal lesion at ostial left anterior descending (white arrow head). **b** Plaque was removed by the directional coronary atherectomy (Atherocut®, NIPRO Corp, Tokyo, Japan) followed by a paclitaxel-coated balloon (SeQuent Please Neo®, B. Braun, Melsungen, Germany). **c** Post-procedural angiographic result was fine. The patient was asymptomatic and scheduled follow-up was planned by the coronary computed tomography (CT) angiography at 12 months after the procedure. The maximum intensity projection (**d**), volume rendering (**e**), and CT-derived fractional flow reserve (FFRCT) (**f**) showed excellent results without any evidence of restenosis (yellow arrow heads). This case highlights an advantage of the “stentless” or “leave nothing behind” strategy in non-invasive anatomical and functional assessment by CT scan at the clinical follow-up

Recently, the DCB in conjunction with the directional coronary atherectomy (DCA) demonstrated excellent clinical outcomes for bifurcation lesions (81% at left main bifurcation) [38]. The percentage plaque area after DCA was 56.3% in this Japanese multicenter registry, and the primary endpoint (TVF at 12 months) and binary restenosis were observed in 10.9% and 2.3%, respectively. This could imply that DCA followed by DCB could be an attractive option for preventing carina shift in large vessel bifurcation lesions (Fig. 2).

#### Diffuse long lesions

Diffuse long lesions may frequently require stenting with long (≥ 60 mm) overlapping DESs which is known as a predictor of adverse events such as restenosis or stent thrombosis [39, 40]. Given the natural step-down of vessel diameter and recent data supporting the safety and efficacy of DCB-only strategy in small vessel lesions, a hybrid approach combining DES (proximal) and DCB (distal) has been proposed for treating de novo diffuse long lesions. For example, the “full metal jacket” with metallic DESs would be undesirable specifically for young patients having diffuse long lesions because it would preclude the possibility of future surgical options. A previous study demonstrated that a DCB-only strategy and a hybrid approach had comparable rates of major adverse cardiovascular events (20.8% vs. 22.7%; *p* = 0.74) and TLR (9.6% vs. 9.3%, *p* = 0.84), respectively [41]. Another recent study also demonstrated that in diffuse lesions (mean lesion length: 44 mm), patients treated with DCB alone or in combination with DES had comparable 3-year clinical outcomes [42]. The HYPER pilot study will evaluate the 12-month clinical outcomes of a hybrid (DES/DCB) approach in 100 patients with diffuse CADs [43].

#### Calcified lesions

Calcified lesion remain as an “Achilles’ heel” of PCI even in the DES era. The following factors contribute to its poor response to DES technologies; (1) stent underexpansion; (2) vessel wall overstretch resulting in medial injury or disruption; (3) damaged polymer coatings; (4) stent fractures; and (5) delayed arterial healing responses. Although the efficacy of the DCB in such lesions has not yet been proven, the DCB-only strategy may be considered as a reasonable therapeutic option if the metallic DES is not expected to be expandable or effective. Calcified nodules or nodular calcifications have been identified as one of the most malignant phenotype of lesion morphologies associated with poor clinical outcomes [44]. Sometimes eruptive calcified tissue is protruding into the lumen beyond the stent struts even after DES implantation. Such a type of lesion is considered to be an extremely high risk for restenosis, and is typically resistant to currently available therapeutic approaches. Although the DCB-only strategy for calcified nodules is still being researched, by eliminating the implantation of metallic prostheses, it may leave additional therapeutic choices.

#### Acute coronary syndrome (ACS)

Only limited data regarding the DCB are available in patients presenting with ACS. The REVELATION trial found that the DCB-only strategy was not inferior to DES in terms of fractional flow reserve at 9 months in 120 patients with ST-segment elevation myocardial infarction (STEMI) undergoing primary PCI [45]. Interestingly, only one case with abrupt closure and one case requiring TLR were observed in the DCB group. In 210 cases of non-ST-segment elevation myocardial infarction (NSTEMI), the recent PEPCAD NSTEMI trial found no difference in TLF between paclitaxel DCB and metallic stents (i.e., BMS or DES) [31]. These findings might support the use of DCB in ACS patients, although a caution is necessary given that these were very carefully selected populations.

In contrast to chronic coronary syndrome (CCS), ACS is caused by the presence of thrombus. The possibility that thrombus could obstruct the distribution of antiproliferative drugs to the vessel wall and underlying tissue is a potential concern. Another concern is acute abrupt vessel closure which occurred in 8.3% of cases when plain old balloon angioplasty (POBA) was used as the default strategy [46]. Dissections, elastic recoil, vasospasm, and thrombus formation are common causes, and these complications must be avoided in order for DCB procedures to be successful. On the other hand, metallic DES was reported to be more frequently associated with incomplete stent apposition and uncovered struts in STEMI than in CCS [47]. Thus, the concept of DCB-only strategy in thrombotic lesions—eliminating metallic prosthesis without compromising the risk of acute abrupt vessel closure and TLR—is potentially appealing, but proof-of-concept studies are obviously required.

#### High-bleeding risk (HBR)

High-bleeding risk (HBR) has recently gained a lot of clinical and academic interest in interventional cardiology, because as many as 64% of Japanese patients undergoing PCI met the Japanese HBR (J-HBR) criteria [48]. In theory, the aforementioned “stentless” or “leave nothing behind” approach with the DCB may offer an advantage in reducing bleeding risk through shorter duration of DAPT over metallic stent implantation. The current Japanese guideline recommend 1–3 months DAPT for CCS patients treated with DCB-only strategy (class IIa, level of evidence B) [49]. The possibility of a shorter DAPT cannot be ruled out given the absence of foreign materials in the coronary artery and the extremely low incidence of acute thrombotic occlusion reported in the previous literatures, even though recent DES trials consistently showed that a very short (1 month) DAPT strategy significantly reduced the risk of major bleeding without compromising the risk of thrombotic events [50–52]. The current instructions for use (IFU) proposed at least 3-month DAPT after the treatment with DCB, whereas the safety of 1-month of DAPT has been shown for CCS patients with small vessel diseases [28, 30]. Appropriate antiplatelet therapy after DCB is an issue to be explored in the future.

## Technical considerations

### Lesion preparation

**Fig. 3:**
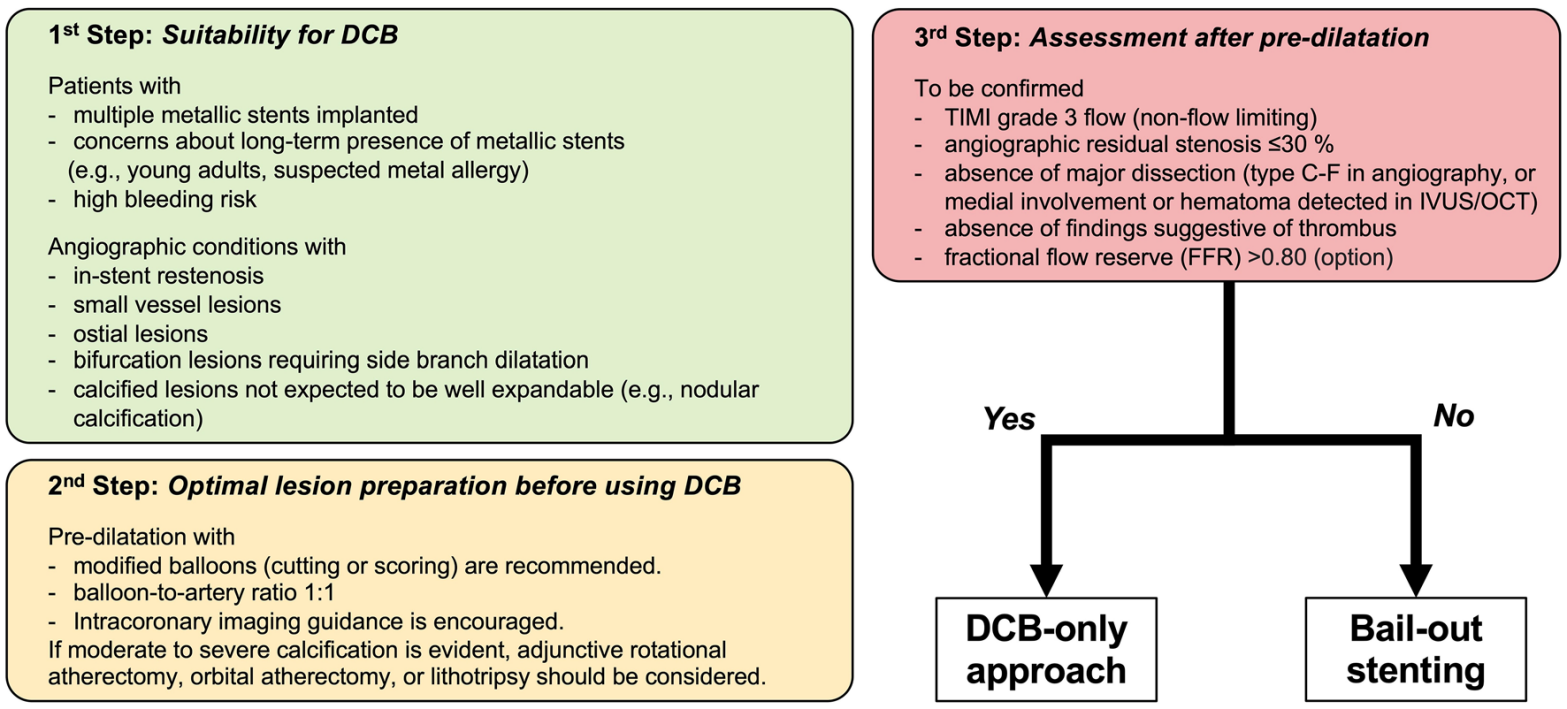
A proposed step-by-step approach for the DCB-only strategy. For possible indication of large vessel (≥ 3.0 mm) lesions, refer to an official statement of the CVIT issued on 1st Dec, 2022. (https://www.cvit.jp/_assets/documents/news/2023/0104.pdf). DCB, drug-coated balloon; IVUS, intravascular ultrasound; OCT, optical coherence tomography; DES, drug-eluting stent

The proposed procedural strategy for the DCB is shown in the Fig. 3. Optimal lesion preparation is of paramount importance to maximize the effect of DCB. Adequate angiographic findings defined as thrombolysis in myocardial infarction (TIMI) grade 3 flow, residual stenosis ≤ 30%, and absence of major dissections (i.e., NHLBI classification type C–E [53]) after pre-dilatation were associated with a lower risk of repeat TLR in ISR lesions [54]. The HOST-ISR-DEB cohort study found that fully optimized procedures with a balloon-to-stent ratio > 0.91, total inflation time > 60 s, and residual stenosis < 20% has a significantly lower incidence of TLF within 2 years than partially or non-optimized procedures [55].

Pre-dilatation with a scoring balloon prior to DCB significantly reduced the incidence of binary angiographic restenosis in a previous randomized trial in patients with DES restenosis (18.5% vs. 32.0%) [56]. More aggressive pre-dilatation or debulking devices, such as rotational atherectomy or excimer laser, should be considered for calcified lesions. Notably, orbital atherectomy and intracoronary lithotripsy are not recommended for stent-related lesions in the IFU reviewed by the Japanese Pharmaceuticals and Medical Devices Agency (PMDA). When major dissections are suspected in angiogram, intravascular ultrasound (IVUS) rather than OCT may better provide the severity of dissections in a safer manner [57]. A non-flow limiting intimal dissection can be left untreated, whereas medial dissection, intramural hematoma, or extramedial injury should be fixed with “bail-out” stenting.

Functional assessment after the lesion preparation may be an option to aid our decision-making for the DCB-only strategy. Aside from angiographic residual stenosis, fractional flow reserve (FFR) of > 0.90 or coronary flow reserve (CFR) of > 2.5 has been identified as a cutoff predicting better clinical outcomes following conventional balloon angioplasty [58, 59]. The advent of the DCB lowered the threshold for FFR to 0.85 or even 0.75 [60–62]. Although the limited data must be acknowledged in this regard, the expert consensus would propose FFR of > 0.80 after lesion preparation as a reasonable cutoff to proceed to the DCB-only strategy.

### Geographic miss

Geographic miss, defined as an area that has injured but is not covered by the radiation source, was considered as a potential substrate of recurrent restenosis (e.g., candy wrapper effect) during the intracoronary radiation (i.e., brachytherapy) era [63, 64]. Geographic miss continued to be a technical element causing unfavorable edge vascular responses (EVR) even in the DES era [65]. Similar to the DCB, geographic miss appeared to be an independent predictor of recurrent ISR [66]. As a result, when using conventional semi-compliant or high-pressure balloons for pre-dilatation, special attention should be paid to the possibility of balloon slippage during inflation. Previous research found that when compared to conventional balloon angioplasty, the modified balloons (i.e., cutting balloon or scoring balloon) were associated with significantly lower rates of subsequent stenting in small vessel lesions [67, 68]. The randomized REDUCE III trial clearly showed that lesion preparation with cutting balloon was associated with larger minimal lumen diameter (MLD) and lower percentage diameter stenosis post-stenting, and subsequent lower rate of restenosis at 7-month follow-up compared to that with conventional balloon [69]. It should be noted that intracoronary imaging guidance (i.e., IVUS) would have minimized the risk of complications while maximizing the achieved MLD. Thus, the cutting or scoring balloons are recommended for better lesion preparation to reduce the risk of balloon slippage or unplanned “bail-out” stenting. Considering a potential mechanical damage to the vessel wall, pre-dilated segments should be thoroughly covered by the DCB.

### Device delivery

Currently available DCB system has a larger profile than that of conventional balloons. As a result, the possibility of system delivery failure to the lesion or drug loss due to friction with irregular vessel surface is of procedural concern. In a retrospective study, adjunctive use of guide extension catheters (GEC) was significantly associated with a lower incidence of TVR within 1 year (9.0% vs. 24.2%), despite the fact that the GEC group had older patients and a higher prevalence of complex lesion subsets [70]. This could encourage the adoption of the GEC to ensure quick and seamless delivery of the DCB system.

## Concerns regarding increased mortality with paclitaxel DCBs

In 2018, Katsanos et al. [71] reported a meta-analysis revealing an increased mortality in patients with lower extremity artery diseases (LEAD) who were treated with paclitaxel stents or balloons. This report raised significant concerns related to the use of paclitaxel DCB in coronary arteries. In the DAEDALUS study, Giacoppo et al. [72] demonstrated that the incidence of all-cause mortality in coronary ISR patients was comparable between paclitaxel DCBs and repeat stenting with DES. Scheller et al. [73] addressed another meta-analysis for de novo CAD patients, showing that paclitaxel DCBs were not associated with an increased mortality at 2 years and had a trend toward a lower all-cause mortality at 3 years when compared to DES. Collectively, such a mortality concern has not yet been identified for CAD patients.

## Future perspectives

The temporary halt in development of the bioresorbable scaffold (BRS) technologies might motivate the community to maintain the “leave nothing behind” concept using the DCB. Because there is only limited data (mostly showing positive signals) for de novo large vessel lesions, we must be cautiously optimistic in order to broaden the clinical indications for this technology. Japan is a unique country where intracoronary imaging techniques are commonly used during the PCI. Therefore, imaging-based criteria for optimal lesion preparation and decision-making for DCB-only strategies must be established. The newer sirolimus-based DCB technologies will certainly draw clinical and academic interests. Despite technical challenges in transferring the drug into the vessel wall, rapamycin-based DCBs are expected to alleviate several safety concerns associated with paclitaxel-based DCBs. Finally, it is clear that further studies with a larger sample size and a longer follow-up period are needed to clarify the potential advantage of the DCB over the gold-standard DES.

## Conclusions

Despite the fact that the DES is the most common and established therapeutic strategy in modern PCI, the use of the DCB is steadily increasing in various clinical settings. The DCB technology, like the BRS, is expected to be a therapeutic approach that facilitates the “leave nothing behind” strategy. However, clinical indications of the DCB other than classical ISR or small vessel disease need be further evaluated. Furthermore, additional scientific evidences for the DCB-only strategy should be discussed alongside the “gold standard” DES for the treatment of de novo CADs.
